# Next Generation Sequencing Detects Premeiotic Errors in Human Oocytes

**DOI:** 10.3390/ijms23020665

**Published:** 2022-01-08

**Authors:** Harita Ghevaria, Sioban SenGupta, Roy Naja, Rabi Odia, Holly Exeter, Paul Serhal, Xavier Viñals Gonzalez, Xuhui Sun, Joy Delhanty

**Affiliations:** 1 Preimplantation Genetics Group, Institute for Women’s Health, University College London (UCL), London WC1E 6HX, UK; h.ghevaria@ucl.ac.uk (H.G.); r.naja@ucl.ac.uk (R.N.); xavier.gonzalez.18@ucl.ac.uk (X.V.G.); xuhui.sun@ucl.ac.uk (X.S.); j.delhanty@ucl.ac.uk (J.D.); 2 Embryology Department, The Centre for Reproductive and Genetic Health, London W1W 5QS, UK; rabi.odia@crgh.co.uk (R.O.); holly.exeter@crgh.co.uk (H.E.); 3 Clinical Department, The Centre for Reproductive and Genetic Health, London W1W 5QS, UK; paul.serhal@crgh.co.uk

**Keywords:** human oocytes, premeiotic aneuploidy, meiosis, metaphase II oocyte and first polar body complex, next generation sequencing

## Abstract

Autosomal aneuploidy is the leading cause of embryonic and foetal death in humans. This arises mainly from errors in meiosis I or II of oogenesis. A largely ignored source of error stems from germinal mosaicism, which leads to premeiotic aneuploidy. Molecular cytogenetic studies employing metaphase fluorescence *in situ* hybridization and comparative genomic hybridisation suggest that premeiotic aneuploidy may affect 10–20% of oocytes overall. Such studies have been criticised on technical grounds. We report here an independent study carried out on unmanipulated oocytes that have been analysed using next generation sequencing (NGS). This study confirms that the incidence of premeiotic aneuploidy in an unselected series of oocytes exceeds 10%. A total of 140 oocytes donated by 42 women gave conclusive results; of these, 124 (88.5%) were euploid. Sixteen out of 140 (11.4%) provided evidence of premeiotic aneuploidy. Of the 140, 112 oocytes were immature (germinal vesicle or metaphase I), of which 10 were aneuploid (8.93%); the remaining 28 were intact metaphase II - first polar body complexes, and six of these were aneuploid (21.4%). Of the 16 aneuploid cells, half contained simple errors (one or two abnormal chromosomes) and half contained complex errors. We conclude that germinal mosaicism leading to premeiotic aneuploidy is a consistent finding affecting at least 10% of unselected oocytes from women undergoing egg collection for a variety of reasons. The importance of premeiotic aneuploidy lies in the fact that, for individual oocytes, it greatly increases the risk of an aneuploid mature oocyte irrespective of maternal age. As such, this may account for some cases of aneuploid conceptions in very young women.

## 1. Introduction

In humans, reproduction is regarded as a highly inefficient process. A major factor lies with the uniquely high frequency of aneuploidy in human gametes and embryos [1,2,3,4]. Autosomal aneuploidy in human gametes arises mostly from errors in meiosis I or II of oogenesis, but mosaicism from errors in early embryogenesis is also a major cause of embryonic death [5,6].

**Figure 1:**
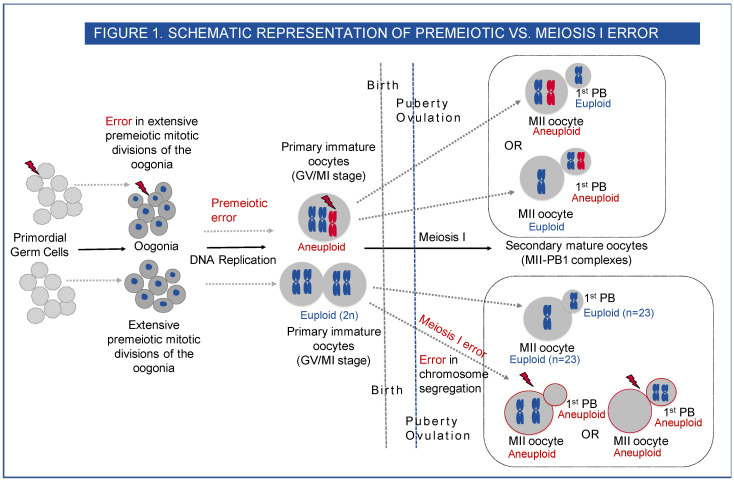
Schematic representation of premeiotic vs. meiosis I error. Human oogenesis begins before birth in the foetus. By the end of the third month of foetal development, the primordial germ cells (PGC) have undergone multiple rounds of mitosis to form oogonia, which undergo premeiotic DNA replication, and the oocytes then enter the prophase I of meiosis I. The oocytes continue through the leptotene, zygotene and pachytene stages of meiotic prophase I. Near the time of birth, all the oocytes have sequentially started the prophase I of meiosis I and are arrested at the diplotene stage. These primary oocytes remain arrested in prophase and are characterised by a nucleus called the germinal vesicle (GV). This stage lasts for many years, and the oocytes do not finish their first meiotic division until after puberty. Premeiotic aneuploidy may arise in the extensive premeiotic mitotic divisions of the oogonia, leading to the gain or loss of chromosome in the primary oocyte at the GV or MI stage (immature oocytes) or mosaic premeiotic aneuploidy may be present in the primordial germ cells, i.e., if a few of the PGCs are aneuploid to start with, they differentiate through mitotic divisions to produce a mix of euploid and aneuploid oogonia. The aneuploid oogonia will then lead to the formation of aneuploid primary oocytes (GV or MI) and on completion of meiosis I, aneuploid secondary metaphase II oocyte—1st PB complexes (MII-PB). At puberty with each monthly cycle, the oocytes sequentially resume meiosis I and proceed through the metaphase I (MI) and anaphase I stages, and the mature gamete is formed after the completion of meiosis I with 23 chromosomes in the metaphase II (MII) oocyte and another 23 chromosomes in the first polar body (1st PB). An error at this stage will lead to aneuploid MII and aneuploid 1st PB (meiosis I error).

However, few studies consider the possible contribution of aneuploidy that exists premeiotically in the human female. The origin of this type of aneuploidy occurs either in the primordial germ cells themselves, a proportion of which may be trisomic or monosomic (gonadal mosaicism) or during the extensive premeiotic mitotic divisions of the oogonia that occur during early foetal life. The term germinal mosaicism covers both types [7]. (Figure 1)

Based upon published cytogenetic analyses carried out on many hundreds of metaphase II oocytes, it is generally considered that the contribution of germinal mosaicism is negligible. This is understandable, since evidence of premeiotic aneuploidy requires either the analysis of immature oocytes (germinal vesicle (GV) or metaphase I (MI)) or the analysis of both the MII oocyte and its corresponding first polar body (1st PB) [8]. These studies suggest that premeiotic aneuploidy may affect 10–20% of oocytes from the population of women undergoing egg collection for a variety of reasons. Earlier investigations employed the fluorescence *in situ* hybridisation (FISH) technique [9,10]. More recently, comparative genomic hybridisation (CGH) was used [11]. In all of these studies, the finding of premeiotic aneuploidy was incidental and not the main focus of the investigation.

In order to systematically investigate the origins of oocyte aneuploidy, we used array comparative genomic hybridisation (aCGH) in the analysis of oocytes unexposed to sperm. The GV and MI stage oocytes that have not completed meiosis I provided direct information on premeiotic errors. Errors seen in the mature metaphase II oocyte and its first PB are expected to be reciprocal, with a loss in one mirrored by a gain in the other, and can be either of chromosome or chromatid origin. Non-reciprocal gains provide evidence of premeiotic aneuploidy. As well as giving an estimate of the frequency of germinal mosaicism, our study also compared the incidence of premeiotic aneuploidy with that of errors arising at meiosis I in the same cohort of women [12] (Figure 1).

Our results provided clear evidence that premeiotic aneuploidy may be detected in 10% of informative oocytes (those that provide information on premeiotic errors—GV, MI and MII-PB complexes), affecting 16% of women that are undergoing egg collection, but who are not necessarily infertile. In cases where the source of the error could be determined, 38% were caused by germinal mosaicism compared with 62% that were the outcome of a meiosis I error [12]. The relative frequencies of these two mechanisms will depend upon the average maternal age of the cohort being investigated.

Following the somewhat surprising findings of our study confirming the relatively common occurrence of premeiotic aneuploidy, suggestions were made that they could be artefactual as a result of damage to the DNA caused by the separation of the MII oocyte from its associated 1st PB and, secondly, that some of those classed as MI may in fact have been MIIs that had lost their PBs. [12] We have now carried out this current study using next generation sequencing (NGS) on a series of oocytes where there was no manipulation of the oocyte. Oocytes were either immature (GV or MI) or were mature with the MII plus its 1st PB tubed together. More specifically, the GVs and MIs were tubed with their ‘zona pellucida intact’ and the MII oocyte plus its first PB (MII+PB) tubed together in a single reaction tube, with an intact zona pellucida prior to NGS analysis. This ensured that the entire DNA content of the oocytes at different maturation stages remained intact and that no DNA was lost during NGS analysis.

## 2. Results

**Figure 2:**
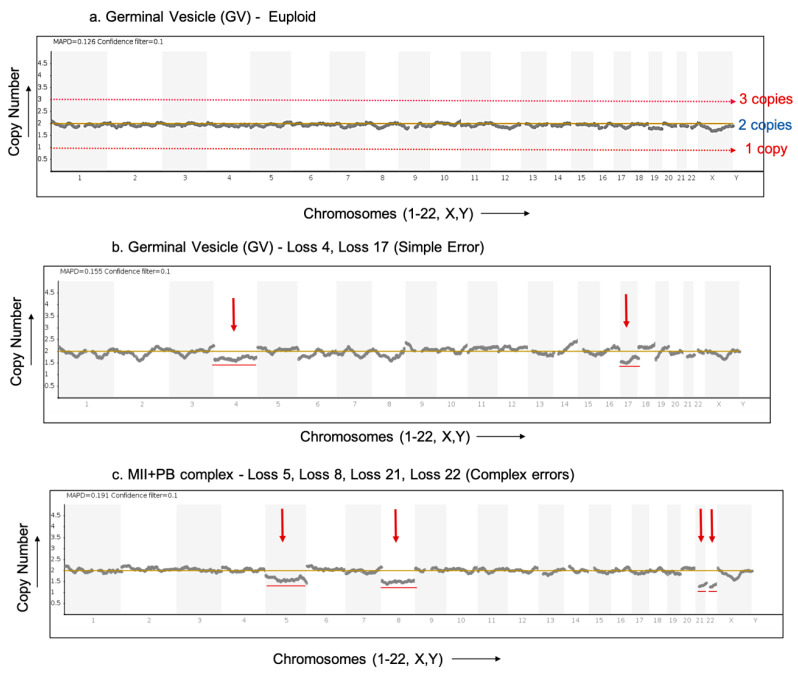
Result profiles of euploid and aneuploid oocytes analysed via NGS. The *X*-axis represents the chromosome number (Chromosome 1–22, X,Y). The *Y*-axis of the NGS graphs represents the chromosome copy numbers. (**a**) NGS profile of a euploid germinal vesicle (GV) stage oocyte with no loss or gain detected. (**b**) NGS profile of an aneuploid germinal vesicle (GV) stage oocyte (donor F) with red arrows indicating loss of chromosomes 4 and 17 (simple error). (**c**) NGS profile of an aneuploid MII+PB complex (donor D) (both cells analysed together in a single reaction) with red arrows indicating loss of chromosomes 5, 8, 21 and 22 (complex errors).

A total of 149 oocytes were donated for research. Overall, 11% oocytes gave evidence of premeiotic (PM) aneuploidy. The DNAs from 147 oocytes (immature and mature) (98.6%) were successfully amplified and subjected to NGS analysis using either Veriseq™ PGS or ReproSeq™ PGS aneuploidy analysis kits and 140 (95.2%) gave conclusive results. These 140 oocytes were donated by 42 women. Seven oocytes gave inconclusive results due to the degradation of oocyte DNA. The average maternal age of 42 women whose oocytes gave conclusive NGS results was 35.33 years (range 23–44 years). A total of 124 (88.5%) oocytes were found to be euploid with no loss or gain of any chromosome (Figure 2a). Overall, 16/140 (11.4%) oocytes showed PM aneuploidy. The 16 aneuploid oocytes with PM aneuploidy were donated by 10 (23%) women. The average age of women whose oocytes showed PM aneuploidy was 35.3 years. All 23 chromosomes were observed to be involved in a PM aneuploidy event in the oocytes tested in this study.

Immature oocytes include GV and MI stage oocytes. A total of 112 immature oocytes were analysed, of which 10 oocytes (8.93%) showed PM errors. The aneuploid immature oocytes included three GVs, five MIs and two GV/MI (where the exact stage of oocyte maturation was not clearly identifiable). Figure 2b shows an aneuploid germinal vesicle (GV) stage oocyte showing the loss of chromosomes 4 and 17.

In total, 28 MII+PB complexes were analysed with both the MII oocyte and 1st PB (intact) in a single reaction. Such MII+PB complexes provide information only on premeiotic errors. Of the 28 MII+PB complexes analysed together, six (21.4%) oocyte complexes showed PM errors. Figure 2c shows an aneuploid MII+PB complex with the loss of chromosomes 5, 8, 21 and 22. In Figure 2b,c, certain aneuploidies show non-integer values on the *Y*-axis (copy number), indicating possible contamination with cumulus cells. In such cases, a copy number variation is seen as a decimal value over a baseline (CN = 2) instead of an integer value. The presence of this contamination tends to mask the true deviation (loss/gain) of the aneuploidy which, in turn, will make the specific gains and losses appear lower (smaller shift) than the expected copy number deviation [13].

The overall summary of the oocytes tested at different maturation stages, along with the numbers of euploid and aneuploid oocytes with PM errors, are shown in Table 1.

The PM errors seen in the oocytes were of two types: simple errors, which are defined as errors involving one to two aneuploidies each, and complex errors, defined as aneuploidies involving three or more distinct chromosomes. In this study, of the 16 aneuploid oocytes with PM errors, eight oocytes showed simple errors and another eight oocytes showed complex errors (Table 2). As shown in Table 2, sixteen oocytes from 10 donors contributed to PM aneuploidy. These donors showed PM aneuploidy in the range of 14% to 50% of oocytes tested. The information with respect to the number of aneuploid oocytes with a PM error along with the total number of oocytes tested for each donor (showing PM errors) is also included.

## 3. Discussion

It is well known that human reproduction is very susceptible to errors and that chromosomal abnormalities make a substantial contribution to implantation failure (both in vivo and in vitro) in humans [14]. This study has directly investigated the premeiotic errors occurring in unmanipulated immature and mature human oocytes using NGS. Premeiotic errors provide evidence of germinal mosaicism.

The analysis of MII oocytes and corresponding 1st PBs by FISH using chromosome specific probes first showed premeiotic errors in terms of the non-reciprocal errors seen in MII-PB complexes. Several studies have since used comprehensive chromosomal analysis methods, such as mCGH, on mature oocytes, obtained as immature or those which have failed to fertilize (unfertilized). These studies revealed variable rates of premeiotic aneuploidy, such as 5.2% [9], 20% [10], 9.5% [15] and 15% [11,16]. Therefore, all molecular cytogenetic methods used, from metaphase FISH, CGH, aCGH and now NGS, show that germinal mosaicism leading to premeiotic aneuploidy is a consistent finding [9,10,11,12]. The detection of germinal mosaicism requires copy number as well as SNP (single nucleotide polymorphism) analysis for accurate detection [8]. Three distinct products of female meiosis (1st PB and 2nd PB, corresponding activated oocytes or fertilized embryos) have been previously analysed using an SNP genotyping bead array, and meiomaps were constructed for obtaining haplotypes that provide information on the mechanisms of meiotically derived aneuploidy and chromosome mis-segregation patterns [17,18]. However, in the case of premeiotically derived errors, SNP-based analysis (for aneuploidy detected through the absence or presence of SNPs from an entire chromosome) alone cannot detect copy number changes if some of the chromatids of the maternal chromosomes involved in the aneuploidy have identical haplotypes, i.e., aneuploidy originating via mitotic nondisjunction caused by mitotic errors during the expansion of the oogonia, as this may be the most common mode of origin [8].

The present study on GVs, MIs and MII+PB complexes (all tubed with their zona pellucida intact), analysed with NGS, reaffirms that the overall incidence is at least 10% of all oocytes [12].

In the present study, sixteen oocytes with premeiotic errors were donated by 10 donors. Amongst the 10 donors, the percentage of oocytes with premeiotic aneuploidy from a single donor ranged between 14 and 50%. During premeiotic mitoses, there is an enormous increase in cell numbers that provides ample opportunity for the occurrence of separate mitotic errors that may affect only a single mature oocyte (Table 2) [8,12]. Donors C and D had over 50% of oocytes affected with premeiotic errors. For each of these donors, there were differences in the aneuploid chromosomes between the oocytes, suggesting that these aneuploidies did not arise as a result of the clonal expansion of a single abnormal cell.

For the purpose of this study, the oocytes collected were unexposed to sperm and unmanipulated prior to NGS analysis. During oocyte processing, by keeping the oocytes with the zona pellucida intact, any loss or gain of chromosomal material that occurred due to premeiotic aneuploidy should have been identified. Additionally, the oocytes were collected and processed in a similar way as would be for PGT-A (preimplantation genetic testing for aneuploidy). The method of NGS used in this study is now the most commonly applied technique that is used to evaluate the whole chromosome and segmental losses and gains in the trophectoderm biopsies of human embryos [19]. This approach requires PCR-based whole genome amplification (WGA) prior to NGS, which limits the ability to perform high-throughput sequencing. Although this sequencing method is sufficient for the detection of aneuploidy in human preimplantation embryos, both the sequencing depth and genome coverage were not sufficient to perform any further analysis of the actual genotype. The coverage depth is typically low (in the order of 0.01× across the genome) and not suitable to call SNPs or indels. It was therefore not possible to obtain haplotype information or detect structural rearrangements that may have provided important information concerning mechanisms that lie behind the generation of premeiotic aneuploidy [8].

Whilst this study has detected the presence of premeiotic aneuploidy in human oocytes (11.4%), it was not possible to have firm evidence as to the mechanism by which it arose. However, the mechanisms by which aneuploidy arose in oocytes with high levels of aneuploidy (50%) may be different from the rest of the donors where a single oocyte was aneuploid (Table 2).

A number of studies have been published describing the origins and mechanisms leading to aneuploidy in ageing human oocytes, arising due to chromosome segregation errors during meiosis I and II, mainly during the two consecutive cell divisions and during the two cell cycle arrests [4,20]. Current views based on recent oocyte studies suggest that less stringent spindle assembly checkpoint (SAC), spindle instability, multipolarity and merotelic attachments during meiosis I contribute to high aneuploidy in both younger and older women [20].

For future work, it is important to understand that the defining feature of premeiotic errors is their origin and timing of error. In order to elucidate the mechanisms underlying the generation of premeiotic aneuploidy, the molecular mechanisms associated with the processes of (i) the migration and differentiation of primordial germ cell PGCs (in the developing ovary) during early embryogenesis and the (ii) passage through multiple rounds of mitotic divisions to form oogonia during the first few months of the foetal gestation period prior to the onset of meiosis must be investigated. Recently, mechanisms of mitotic chromosome mis-segregation have been elucidated in cleavage- and blastocyst-stage human embryos through genome-wide association analysis. For example, the altered expression of a gene variant of PLK4 gene, a kinase-encoding gene that has a critical role in centrosome duplication during mitosis has been shown to permit the tripolar spindle segregation that leads to extensive aneuploidy in the embryo [21,22].

The estimated chance of achieving a live birth from a clinically recognized natural pregnancy in young fertile couples (age < 31 years) ranges somewhere between 22 and 40%. About 30% of embryos do not implant successfully and cytogenetic studies reveal that most of the pregnancy losses before or after conception are due to chromosomal errors in gametes and embryos [23]. Chromosomal abnormalities may arise in humans premeiotically (either present in the PGCs or in the premeiotic mitoses of the PGCs) or during gametogenesis (meiotic divisions I and/or II). Abnormalities can also arise at fertilisation or after fertilisation (during embryogenesis). It is generally accepted that aneuploidy mainly originates from errors in female meiosis [5]. However, post-zygotic errors (leading to chromosome mosaicism) also contribute to both gametic and embryonic demise [2,3]. Among the risk factors associated with the incidence of chromosomal abnormalities, increasing maternal age is thought to be a well-established risk factor [24].

The importance of premeiotic aneuploidy lies in the fact that, for individual oocytes, it greatly increases the risk of an aneuploid mature oocyte irrespective of maternal age. As such, this may account for some cases of aneuploid conceptions in very young women [8,25]. Crucially, premeiotic aneuploidy will not be detected by metaphase II analysis alone; results from the corresponding 1st PB are also required. Hence, many studies fail to take its existence into account [25]. The wider implication of the findings of the present study emphasizes the fact that premeiotic aneuploidy is one of the age-independent mechanisms that predispose women to produce aneuploid gametes and may be linked to their sub-fertility or infertility. However, the mechanisms by which the aneuploidy arises remains to be elucidated [8].

## 4. Materials and Methods

### 4.1. Patient Details

A total of 149 human oocytes were collected at the Centre for Reproductive and Genetic Health (CRGH), London (UK), after obtaining appropriate research consent. The oocytes were collected between the years 2015 and 2020. The oocytes were donated by 42 women between the ages of 25 and 44 years (mean ± SD of maternal age 35 ± 3.4 years). The donors providing the oocytes were either undergoing routine fertility treatments, which involved conventional IVF (in vitro fertilisation)/ICSI (intracytoplasmic sperm injection) for fresh embryo transfer or embryo freezing, preimplantation genetic testing for various indications (aneuploidy -PGT-A; monogenic/single gene defects -PGT-M; structural rearrangements PGT-SR) or undergoing oocyte cryopreservation for medical or social reasons. The oocytes obtained were unexposed to sperm and were at various oocyte maturation stages.

### 4.2. Oocyte Details and Processing

**Figure 3:**
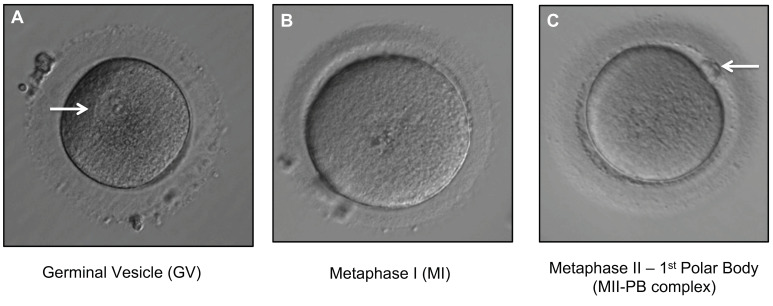
Different stages of oocyte maturation. All oocytes were denuded (removal of the surrounding layer of cumulus cells). (**A**) Immature GV oocyte. Arrow indicates a germinal vesicle (visible nucleus) with a prominent single nucleolus (400× magnification). (**B**) Immature MI oocyte showing absence of germinal vesicle and no 1st PB (400× magnification). (**C**) MII-PB complex (mature MII oocyte with 1st PB). Arrow indicates 1st PB (400× magnification).

In total, 112 immature oocytes including germinal vesicles (GV), metaphase I (MI) stage oocytes and 28 mature oocyte complexes, metaphase II–1st polar body complexes (MII+PB) were collected (Figure 3). Transvaginal oocyte retrieval was performed at 37 h post human chorionic gonadotropin (HCG) (or comparable trigger injection). For intracytoplasmic sperm injection (ICSI) cycles, the removal of cumulus cells was performed 39–40 h post trigger by exposure to Cumulase (Origio Specialty Pharma, Denmark) and oocytes were graded depending on their maturation (germinal vesicle, metaphase I, or metaphase II). Post denudation, oocytes were placed in fertilisation media (Origio, Denmark) and cultured at 37 °C, 5.5% CO_2_, 5% O_2_ and 87.5% N_2_. Immature oocytes were reassessed for maturation at 41 h post HCG prior to ICSI. Thus, only the oocytes that remained immature at this point were deemed unsuitable for clinical use and, therefore, were included in this study. The immature oocytes were individually washed in multiple droplets of PBS/BSA buffer and transferred into a sterile 0.2 μL PCR tube with a minimal amount of PBS/0.1% BSA (Igenomix, UK). For oocytes at the GV or MI stage, the zona pellucida was not removed post oocyte washing. The oocytes at the MII+PB complex stage, the MII oocyte plus the 1st polar body (referred to as MII+PB complex in the results section) were tubed together (with intact zona pellucida) in the same PCR analysis tube. All samples tubed were placed on a PCR rack and stored at −80 °C until subsequent aneuploidy analysis via next generation sequencing (NGS). Once the sample size was reached for a given batch, the PCR racks containing the oocytes were placed in a cooler box, sandwiched between two ice packs, sealed and sent via overnight courier which arrived at the genetic lab the following morning.

A total of 145 oocytes were subjected to aneuploidy analysis using the NGS-based Reproseq PGS kit (Thermo Fisher) and the remaining four oocytes underwent aneuploidy analysis using the NGS-based VeriSeq PGS kit (Illumina, San Diego CA, USA), analysed by Igenomix Laboratory (Guildford, UK).

### 4.3. VeriSeq-NGS Protocol (Illumina)

For whole genome amplification (WGA), the oocyte samples were first lysed, and genomic DNA was randomly fragmented and amplified using the SurePlex DNA Amplification System (Illumina, Inc., San Diego, CA, USA), according to the manufacturer’s protocol. Library preparation: WGA SurePlex template products were purified using the Zymo DNA Clean & Concentrator (Zymo Research Corporation, Irvine, CA, USA) and quantified using the Qubit^®^ dsDNA HS Assay Kit (Life Technologies Corporation, Grand Island, NY, USA). One nanogram of quantified dsDNA template at 0.2 ng/μL was added to 5 μL of amplicon tagmentation mixture (ATM) and 10 μL of tagmentation DNA buffer (TD). The tagmentation step was carried out at 55 °C for 5 min and held at 10°C. The resulting tagmented mixture was neutralized by adding 5 μL of proprietary neutralization buffer (NT). Post-homogenization, the tagmentation plate was held at room temperature for 5 min. The tagmented DNA was amplified via a limited-cycle PCR programme (one cycle of 72 °C for 3 min, 95 °C for 30 s, followed by 12 cycles of 95 °C for 10 s, 55 °C for 30 s and 72 °C for 30 s, one cycle at 72 °C for 30 s, followed by a hold at 4 °C) after the adding of 5 μL of index 1 (i7), 5 μL of index 2 (i5) and 15 μL of Nextera PCR Master Mix (NPM) to each well.

PCR product clean-up used AMPure XP beads (A63881, Beckam Coulter, Brea, CA, USA) to purify the library DNA with no salt carryover, providing a size selection step that removes short library fragments, including index 1 (i7) and index 2 (i5), from the population. Using a multichannel pipette, 45 μL of the PCR product was transferred to 96-well storage plates containing 45 μL of AMPure XP beads. Sealed plates were mixed using a microplate shaker at 1800 rpm for 2 min, then incubated at room temperature without shaking for 5 min. Thereafter, the plate was placed on a magnetic stand (AM10027, Life Technology) for 2 min or until the supernatant cleared. While the plates were kept on the magnetic stand, the magnetic beads were washed twice with 200 μL of freshly prepared 80% ethanol. Purified libraries were eluted with 50 μL of the Nextera XT Resuspension Buffer.

Single-end, dual index 36 base pair read (1 × 36 donor insemination) sequencing was performed following the Illumina v2 chemistry workflow on a MiSeq System (Illumina, Inc.), using the MiSeq Reagent Kit v2 kit (Illumina, Inc.), which contains the ready-to-load on-board clustering and sequencing by synthesis (SBS) chemistry reagents. The sequencing data derived from the MiSeq Reporter Software were then uploaded and analysed using BlueFuse Multi v3.0 for NGS (Illumina, Inc.). The quality control acceptance criteria were the number of total reads > 700,000 with a number of reads passing the filter > 500,000, and the DLR (derivative log ratio) at <0.4. The quality metrics for all the four oocytes analysed via Veriseq-NGS were within the acceptable limits.

### 4.4. Reproseq-NGS Protocol (Thermo Fisher)

DNA extraction and whole-genome amplification (WGA) was performed using an Ion Reproseq PGS kit (Thermo Fisher). The oocyte samples were tubed in 2.5 μL of 1X PBS/0.1%BSA, treated with 5 μL of extraction enzyme master mix and incubated at 75 °C for 10 min, followed by incubation at 95 °C for 4 min. Extracted genomic DNA was pre-amplified with 5 ul of pre-amplification master mix and incubated according to the following program: 1 cycle at 95 °C for 2 min and 12 cycles at 95 °C for 15 s, 15 °C for 50 s, 25 °C for 40 s, 35 °C for 30 s, 65 °C for 40 s, 75 °C for 40 s, and holding at 4 °C. Subsequently, 30 μL of Amplification master mix and 5 μL of Ion SingleSeq Barcode Adaptor were added to each sample. Library amplification was performed with the following PCR program: 1 cycle at 95 °C for 3 min, 4 cycles at 95 °C for 20 s, 50 °C for 25 s, 72 °C for 40 s, 12 cycles at 95 °C for 20 s, 72 °C for 55 s, and holding at 4 °C. Libraries were then pooled, purified with AMPure XP beads (Beckman Coulter, Brea, CA, USA), quantified using the Qubit dsDNA High Sensitivity Assay kit (Life Technologies, Carlsbad, CA, USA) and diluted to the final concentration of 80 pM. Template preparation and chip loading was performed using the Ion Chef system (Thermo Fisher) according to manufacturer’s instructions. In each batch, 23 samples combined with one positive control sample were sequenced together in one Ion 520™ Chip. The chip was then loaded and sequenced on an Ion S5 Sequencer^TM^ (Thermo Fisher). The sequencing data were then transferred from the Ion S5 “Torrent Suite” software v5.10.1 to the Ion Reporter^TM^ software v5.4 (Thermo Fisher) for aneuploidy “calling”. This software uses the ‘ReproSeq PGS w1.1′ workflow (low-pass whole-genome aneuploidy workflow, which detects aneuploidies and whole chromosome and sub-chromosome abnormalities (~10 Mb up to a whole chromosome) from a single whole-genome sample with low coverage (0.01× across the genome)). The normalization was performed using the informatics baseline Reproseq Low Coverage Whole-Genome Baseline (v5.2) generated from multiple normal samples. The Reproseq PGS w1.1 is designed to detect and call aneuploidy events with integer ploidy values. In addition, the aneuploidy “calls” were verified by an in-house-developed algorithm that uses machine learning.

The resulting NGS graphs from both sequencing platforms show chromosomal gains associated with a copy number ≥ 3 and chromosomal losses with a copy number ≤ 1. Where non-integer ploidy values were detected, this was attributed to cumulus cell contamination. Aneuploidy calls were generated by the NGS analyses software (BlueFuse Multi Software Version v3.0 (Illumina)/Ion Reporter™ v5.4 software (Thermo Fisher)) and were also ‘assessed manually’ by ‘two independent scientists’.

The quality control acceptance criteria were the total number of mapped reads of >60,000 and an MAPD (median of the absolute values of all pairwise differences) value of <0.3. The quality metrics for 132/136 oocytes were within the acceptable range. However, for four samples, MAPD results were above the cut-off values. In two oocytes with complex aneuploidies (many step changes), a slightly high MAPD value was detected; however, the results were deemed suitable for manual interpretation by two independent scientists. In the other two oocytes with single aneuploidies, the aneuploidy was called by the software algorithm and interpreted manually.

### 4.5. Validation

Single cells from 8 commercial fibroblast cell lines (Coriell Institute, Camden, NJ, USA), three diluted genomic DNA samples (7.5 pg) (in order to mimic a single cell/blastomere), with known karyotypes, along with a CEPH positive control and a negative control, were included in the validation. The NGS results from single cells and diluted genomic DNA samples had a 100% concordance with the expected chromosome abnormalities/karyotypes. The karyotypes of the cell lines and genomic DNA samples included in the validation are given in Table 3.

### 4.6. Oocyte Analysis and Interpretation of Data

Immature oocytes—GVs and MIs: A euploid GV or MI is expected to have 2 copies of each autosome and 2 ‘X’ chromosomes, i.e., GV and MI oocytes are normally diploid (2n) (Figure 2a). Any chromosome loss or gain is evidence for a premeiotic error.

Mature oocytes—MII+PB complexes: A euploid MII or 1st PB is expected to have 2 sister chromatids of each autosome and of the X chromosome. Therefore, MII and 1st PB when analysed together (MII+PB), the complex altogether is overall diploid. Any chromosome loss or gain is evidence for a premeiotic error.

## Figures and Tables

**Table 1:** Overall summary of NGS results from immature and mature oocyte complexes.

Stage of Oocyte Maturation	Total No. of Oocytes with Conclusive Results	No. of Euploid Oocytes Euploid	No. of Oocytes with Premeiotic Errors	Percentage (%) of Oocytes with Premeiotic Error
Germinal vesicle (GV)	73	70	3	4.11
Metaphase I (MI)	35	30	5	14.29
GV/MI	4	2	2	50
Metaphase II—first polar body (MII+PB)	28	22	6	21.43
Totals	140	124	16	11.43

**Table 2:** Detailed NGS results of aneuploid GV, MI and MII+PB complexes showing premeiotic errors.

Donor ID	Maternal Age (years)	Stage of Oocyte Maturation	Chromosome Aneuploidies (Premeiotic) Detected by NGS	Type of Error	No. of Aneuploid Oocytes/Total No. Oocytes Tested
(A)	35	MI	Gain of 2	Simple error	1/4 (25%)
(B)	32	GV/MI	Gain of 4	Simple error	2/5 (40%)
		GV/MI	Loss of 19	Simple error	
(C)	38	MII+PB	Gain of 4, 7, 8, 10, 16, 21 Loss of 18	Complex errors	3/6 (50%)
		MII+PB	Gain of 16, Loss of 8, 22	Complex errors	
(D)	34	MII+PB	Loss of 22	Simple error	3/6 (50%)
		MII+PB	Gain of 12, Loss of 1, 2, 14	Complex errors	
		MII+PB	Loss of 5, 8, 21, 22	Complex errors	
(E)	36	MII+PB	Gain of 20	Simple error	1/7 (14%)
(F)	35	GV	Loss of 4, 17	Simple error	3/12 (25%)
		GV	Loss of 5 (partial), 6, 16, 17	Complex errors	
		GV	Loss of 3 (partial), 7, 12, 13 (partial), X	Complex errors	
(G)	33	MI	Gain of 1, 6, 7, 9, 11, 12, 16, 18, 19, 21, X	Complex errors	1/2 (50%)
(H)	37	MI	Loss of X	Simple error	1/4 (25%)
(I)	36	MI	Gain of 5	Simple error	1/6 (16%)
(J)	36	MI	Gain of 9 Loss of 5, 20, 22	Complex errors	1/5 (20%)

**Table 3:** Validation of NGS methodology.

Sample	Karyotype
Single cell	47, XXX
Single cell	47, XXY
Single cell	47, XYY
Single cell	47, XX, + 13
Single cell	47, XY, + 18
Single cell	47, XX, + 21
Single cell	46, XX
Single cell	46, XY
7.5 pg genomic DNA	47, XX, + 22
7.5 pg genomic DNA	47, XX, + 20
7.5 pg genomic DNA	48, XY, + 2, + 21

## Data Availability

Not applicable.
